# Association of Homocysteine, Methionine, and *MTHFR* 677C>T Polymorphism With Rate of Cardiovascular Multimorbidity Development in Older Adults in Sweden

**DOI:** 10.1001/jamanetworkopen.2020.5316

**Published:** 2020-05-20

**Authors:** Amaia Calderón-Larrañaga, Marguerita Saadeh, Babak Hooshmand, Helga Refsum, A. David Smith, Alessandra Marengoni, Davide L. Vetrano

**Affiliations:** 1 Aging Research Center, Department of Neurobiology, Care Sciences and Society, Karolinska Institutet, Solna, Sweden; 2 Department of Neurology, Ulm University Hospital, Ulm, Germany; 3 Department of Pharmacology, University of Oxford, Oxford, United Kingdom; 4 Department of Nutrition, Institute of Basic Medical Sciences, University of Oslo, Oslo, Norway; 5 Department of Clinical and Experimental Sciences, University of Brescia, Brescia, Italy; 6 Department of Geriatrics, Fondazione Policlinico “A. Gemelli” IRCCS and Catholic University of Rome, Rome, Italy

## Abstract

**Importance:**

Strong evidence links high total serum homocysteine (tHcy) and low methionine (Met) levels with higher risk of ischemic disease, but other cardiovascular (CV) diseases may also be associated with their pleiotropic effects.

**Objectives:**

To investigate the association of serum concentrations of tHcy and Met with the rate of CV multimorbidity development in older adults and to explore the role of methylenetetrahydrofolate reductase (*MTHFR*) 677C>T polymorphism in this association.

**Design, Setting, and Participants:**

The Swedish National Study on Aging and Care in Kungsholmen is a cohort study of randomly selected individuals aged 60 years or older. The present study included data on 1969 individuals with complete information and without CV diseases at baseline, collected from the baseline examination (2001-2004) to the fourth follow-up (2013-2016). Data analysis was conducted from January to May 2019.

**Exposures:**

Concentrations of tHcy and Met were measured from nonfasting venous blood samples. The Met:tHcy ratio was considered a possible indicator of methylation activity. *MTHFR* status was dichotomized as any T carriers vs noncarriers.

**Main Outcome and Measures:**

The number of CV diseases at each wave was ascertained based on medical interviews and records, laboratory test results, and drug data. Linear mixed models were used to study the association of baseline tHcy and Met levels and the rate of CV multimorbidity development, adjusting for sociodemographic characteristics, CV risk factors, chronic disease burden, and drug use.

**Results:**

Of 1969 participants, most were women (1261 [64.0%]), with a mean (SD) age of 70.9 (9.8) years; 1703 participants (86.6%) had at least a high school level of education. Baseline measurements of serum tHcy, Met, and the Met:tHcy ratio were associated with the rate of CV disease accumulation (tHcy: β = 0.023 per year; 95% CI, 0.015 to 0.030; *P* < .001; Met: β = −0.007 per year; 95% CI, −0.013 to −0.001; *P* = .02; Met:tHcy ratio: β = −0.017 per year; 95% CI, −0.023 to −0.011; *P* < .001). The association between low Met concentrations and the rate of CV multimorbidity development was restricted to the group with CT/TT alleles of *MTHFR* (β = 0.023 per year; 95% CI, 0.006 to 0.041; *P* = .009). Results remained largely significant when individual CV diseases were removed from the total count 1 at a time (eg, ischemic heart disease, tHcy: β = 0.023 per year; 95% CI, 0.013 to 0.027; *P* < .001; Met: β = −0.006 per year; 95% CI, −0.011 to −0.0003; *P* = .04; Met:tHcy ratio: β = −0.015 per year; 95% CI, −0.020 to −0.009; *P* < .001).

**Conclusions and Relevance:**

In this study, high tHcy and low Met levels were associated with faster CV multimorbidity development in older age. The interactive association of Met concentrations and *MTHFR* polymorphism, together with the association found for the Met:tHcy ratio, point toward the relevance of impaired methylation in the pathogenesis of CV aging.

## Key Points

**Question:**

Are serum concentrations of homocysteine and methionine associated with the rate of cardiovascular multimorbidity development in older adults?

**Findings:**

In this cohort study of 1969 older adults in Sweden, high homocysteine levels, low methionine levels, and a low methionine to homocysteine ratio were associated with faster development of cardiovascular multimorbidity. The *MTHFR* 677C>T polymorphism was further associated with accelerated cardiovascular multimorbidity development in participants with a low methionine concentration.

**Meaning:**

The findings of this study suggest that these biomarkers may have a potentially meaningful role of in the pathogenesis of cardiovascular aging, possibly through impaired methylation activity.

## Teaser

This cohort study investigates the association of serum concentrations of homocysteine and methionine with the rate of cardiovascular multimorbidity development in older adults and explores the role of methylenetetrahydrofolate reductase (*MTHFR*) 677C>T polymorphism in this association.

## Introduction

Most age-related cardiovascular (CV) diseases share common underlying biological mechanisms. Telomere attrition,^1^ epigenetic modifications,^2^ defects in autophagy or mitophagy,^3^ and cell senescence^4^ eventually contribute to a pro-inflammatory environment that is common in different disorders, such as atherosclerosis, cardiomyopathies, heart failure, ischemic heart disease, and stroke. The rate at which multiple CV diseases, ie, CV multimorbidity, accumulate is a marker of CV aging.^5^ Thus, the identification of cellular and molecular biomarkers of accelerated CV multimorbidity development may be helpful for secondary prevention and prognostic counselling.

Homocysteine, an amino acid generated via demethylation of dietary methionine, is associated with atherosclerosis and its complications, such as myocardial infarction and stroke.^6,7^ In fact, high concentrations of total homocysteine (tHcy) can lead to endothelial cell damage, impaired vascular compliance, and alterations of hemostasis.^8^ Besides an unhealthy lifestyle, poor diet, impaired renal function, and intake of certain drugs,^9^ moderate increases in tHcy have been associated with a 677C>T variant in the gene coding for methylenetetrahydrofolate reductase (*MTHFR*; OMIM 607093).^10^

Methionine (Met), obtained both from diet and from the remethylation pathway of tHcy metabolism, is an essential amino acid functioning as a precursor for cysteine and glutathione, 2 major antioxidants. Studies in mice suggest that low Met availability affects the development of atherosclerotic lesions and the regulation of inflammatory response and hepatic cholesterol metabolism,^11,12^ but its role in CV diseases is unclear. One study found an association between low blood Met levels and incident myocardial infarction^13^; another study corroborated this association prospectively but only in subjects with high low-density lipoprotein (LDL) cholesterol levels^14^; a third study showed no such association.^15^ A positive association between low Met concentration and venous thrombosis risk has also been reported.^16^

High tHcy and low Met blood concentrations could have antagonistic pleiotropic effects on CV aging, not only via atherosclerosis, the most studied mechanism, but also through other pathways, such as lipid metabolism, DNA synthesis and repair, oxidative damage, development of inflammation, and telomere attrition.^17^ Therefore, it is plausible that tHcy and Met may play a role in multiple physiologic processes underlying CV aging, including left ventricular wall thickening, increased left atrial size, and vascular intimal thickening, stiffening, and fibrosis.^18^ To our knowledge, no prior study has examined the association of tHcy and Met with multiple CV diseases as a group or with the rate at which these disorders accumulate.

We aimed to investigate the association of serum concentrations of tHcy and Met with the rate of CV multimorbidity development in older adults. We further intended to explore the role of *MTHFR* 677C>T polymorphism in this association.

## Methods

### Data Collection

**Figure 1.**
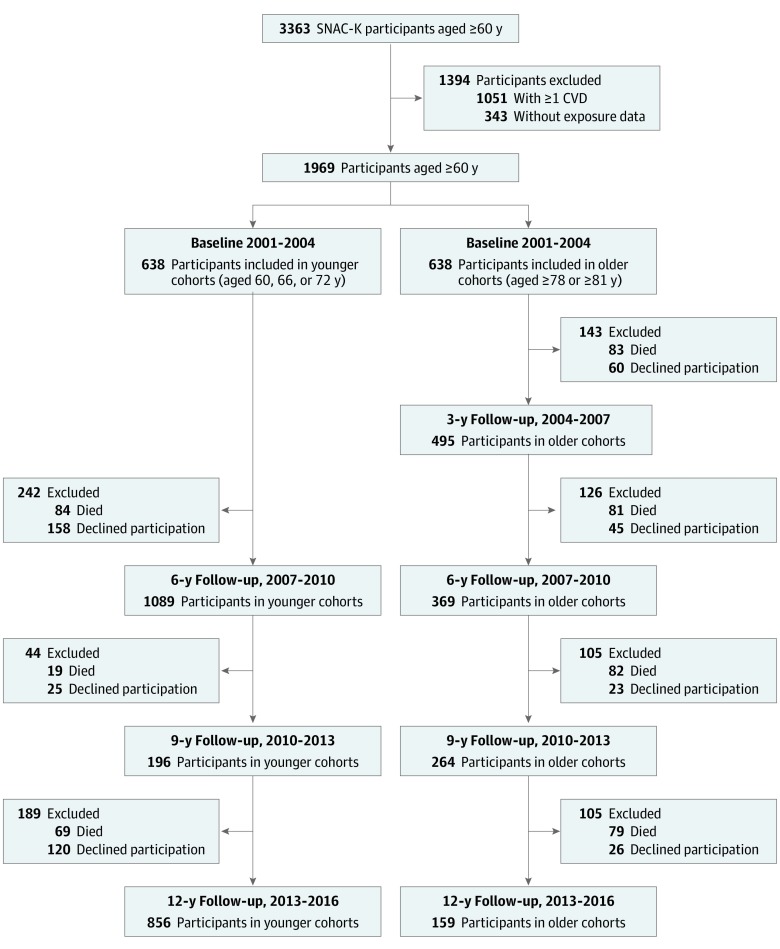
Population Flow Chart for Baseline and First 4 Follow-up Assessments CVD indicates cardiovascular disease; SNAC-K indicates Swedish National Study on Aging and Care in Kungsholmen.

This research is based on data from the Swedish National Study on Aging and Care in Kungsholmen (SNAC-K). This is a community-based longitudinal study of randomly selected individuals aged 60 years or older living at home or in institutions in the Kungsholmen district of Stockholm between 2001 and 2004. Of 4590 eligible participants, 3363 (73.3%) participated in the baseline examination. Participants underwent extensive clinical examinations, interviews, and assessments by physicians, nurses, and psychologists. Data on sociodemographic characteristics, medical history, drug use, laboratory test results, and cognitive function were collected according to structured protocols. Data on medical history and vital status were also obtained by linking SNAC-K data with the National Patient Register and the Swedish Cause of Death Register. Since baseline, SNAC-K participants have been followed up regularly, every 6 years for the cohorts younger than 78 years and every 3 years for those aged 78 years or older. This study included data from baseline and the first 4 follow-ups (Figure 1). In the present study, we excluded 1051 individuals (31.3%) with at least 1 CV disease at baseline plus 343 individuals (10.2%) with missing data on the exposures, leaving 1969 participants (58.5%) in the study cohort. SNAC-K baseline and follow-up protocols have been approved by the Ethics Committee at Karolinska Institutet and the Regional Ethics Review Board in Stockholm, and written informed consent was obtained from participants or their next of kin for those with cognitive impairment.

### Outcome Variable

Chronic diseases were coded using the *International Statistical Classification of Diseases and Related Health Problems, Tenth Revision *(*ICD-10*) and further categorized according to a comprehensive list proposed by Calderon-Larrañaga et al.^19^ For this study, the following CV diseases were considered: ischemic heart disease, stroke, heart failure, atrial fibrillation, cardiac valve diseases, bradycardias and conduction disorders, peripheral artery disease, and other CV diseases (eTable 1A in the Supplement). In addition to the clinical diagnoses made by SNAC-K physicians (based on participant self-report, medical records, inpatient and outpatient registers, anamnestic details, and information gathered from participants’ proxies), complementary clinical and drug-related parameters were used for certain conditions (eTable 1B in the Supplement). Considering the primarily chronic nature of the cardiovascular diseases under study, once detected in any data source, the disease remained present in all following waves. The sum of these diseases at each wave composed the CV multimorbidity.

### Biomarkers

Nonfasting venous blood samples were taken from SNAC-K participants at baseline, and the serum was stored at −80 °C for 10 to 12 years. The concentrations of tHcy and Met were measured using tandem mass spectrometry at the Department of Pharmacology, University of Oxford.^20^ Interassay coefficients of variation were 4.4% for tHcy and 4.0% for Met. The Met:tHcy ratio was considered a possible indicator of methylation activity, with higher ratios indicating higher methylation activity.^21^

Genotyping was performed using matrix-assisted laser desorption/ionization time-of-flight analysis on the MassARRAY platform (Aegna Bioscience) at the Mutation Analysis Facility, Karolinska Institutet. Successful genotyping call rate for rs1801133 (ie, *MTHFR* variation 677C>T) was 94%.

### Covariates

Several covariates were measured at baseline, as follows: age, sex, highest level of formal education (ie, elementary school, high school, or university), use of any type of antihypertensive drug, use of any type of B vitamin supplement, hypertension (based on self-report, medical records, or a SNAC-K blood pressure measurement ≥140/90 mm Hg), diabetes (based on self-report, medical records, SNAC-K glycated hemoglobin measurement ≥6.5% [to convert to proportion of total hemoglobin, multiply by 0.01], or use of antidiabetic drugs), hypercholesterolemia (based on self-report, medical records, or a SNAC-K serum total cholesterol measurement ≥240.2 mg/dL [to convert to millimoles per liter, multiply by 0.0259]), chronic kidney disease (based on self-report, medical records, SNAC-K glomerular filtration rate measurement <60 mL/min/1.73m^2^ according to the Chronic Kidney Disease Epidemiology Collaboration equation), total number of drugs, physical activity (ie, inadequate, health-enhancing, and fitness-enhancing), body mass index (BMI, calculated as weight in kilograms divided by height in meters squared; in 4 groups, as follows: <18.5, 18.5-24.9, 25.0-29.9, and ≥30.0), smoking (ie, never, former, or currently), alcohol consumption (ie, never or occasional, light or moderate, or heavy), serum C-reactive protein (CRP) concentration (ie, ≤0.5 mg/dL and >0.5 mg/dL [to convert to milligrams per liter, multiply by 10]), and time to death or dropout.

### Statistical Analysis

The main outcome variable was the rate of CV multimorbidity development during the 12-year follow-up period. Linear mixed models were used to estimate β coefficients and 95% CIs for the association between baseline concentrations of the biomarkers and the rate of CV multimorbidity development. To measure the association between the exposures of interest and the mean annual increase in the number of CV diseases, interactions between follow-up time and biomarker concentrations were included as fixed effects.^22,23^ Random effects were defined for the intercept and time of follow-up; unstructured covariance was assumed. The exposures (ie, tHcy and Met concentrations) were operationalized both as a standardized continuous variables (*z *scores) and according to tertiles. We created an indicator variable with 4 mutually exclusive categories by cross-classifying individuals’ concentrations of tHcy or Met (ie, high vs low according to medians) and their *MTHFR* categories (ie, CC vs CT/TT) to further explore the combined association of both exposures with the outcome. We dichotomized *MTHFR* status (ie, any T carriers vs noncarriers) to increase group size and thus maintain statistical power. Models were adjusted for sex, age, and education in the minimally adjusted model and for other potential confounders, such as smoking, alcohol consumption, physical activity, BMI, CRP level, number of drugs, dyslipidemia, diabetes, hypertension, chronic kidney disease, use of antihypertensive drugs, use of B vitamin supplements, and time of death or dropout in the fully adjusted model. Confounder selection was evidence informed. In humans, serum concentrations of tHcy are mainly determined by B vitamin levels (ie, folate and B_12_) and renal function, and lifestyle behaviors are among the major associated factors.^9^ Dyslipidemia and CRP level are 2 biomarkers that strongly and independently predict systemic atherosclerosis.^24^ Cardiovascular risk factors have also been included as potential confounding or mediating factors in previous studies.^14^

In a sensitivity analysis, to evaluate whether the associations between baseline concentrations of the biomarkers and the rate of CV multimorbidity development were driven by specific chronic conditions either at baseline or at follow-ups, we reran the models after removing the 8 CV diseases, 1 at a time, from the original count of CV diseases. Analyses were performed using Stata version 15 (StataCorp). Statistical significance was set at *P* < .05, and all tests were 2-tailed.

## Results

The study population consisted of 1969 individuals without any CV disease at baseline, of whom 1261 (64.0%) were women. The mean (SD) age of the study population was 70.9 (9.8) years, and 1703 participants (86.6%) had at least a high school level of education. Most had normal weight (851 [44.5%]) and performed health-enhancing physical activity (995 [50.5%]) (eTable 2 in the Supplement). More than half had hypertension (1342 [68.2%]) and dyslipidemia (994 [50.5%]), but proportions were lower for diabetes (123 [6.3%]) and chronic kidney disease (529 [26.9%]). Nearly one-fifth of the study population used antihypertensive drugs (367 [18.7%]), and 202 (10.3%) used B vitamin supplements. The mean (SD) number of drugs taken at baseline was 2.9 (2.8). Differences between the study population and the entire SNAC-K population can be found in eTable 2 in the Supplement.

The median (interquartile range [IQR]) baseline concentrations were 1.68 (1.38-2.08) mg/dL for tHcy (to convert to micromoles per liter, multiply by 7.397) and 0.35 (0.30-0.40) mg/dL for Met (to convert to micromoles per liter, multiply by 67.02). A significantly higher median (IQR) concentration of tHcy and a lower median (IQR) Met:tHcy ratio were found in the higher age group vs the lower age group (tHCy: 1.596 [1.325-1.920] mg/dL vs 1.893 [1.542-2.420] mg/dL; *P* < .001; Met:tHcy ratio: 2.0 [1.6-2.6] vs 1.6 [1.2-2.0]; *P* < .001), in men vs women (tHCy: 1.785 [1.515-2.164] mg/dL vs 1.623 [1.312-2.042] mg/dL; *P* < .001; Met:tHcy ratio: 1.8 [1.4-2.3] vs 1.9 [1.5-2.5]; *P* < .001), in those with only elementary education vs with a high school or university education (tHCy: 1.893 [1.555-2.393] mg/dL vs 1.704 [1.393-2.137] mg/dL vs 1.596 [1.339-1.920] mg/dL; *P* < .001; Met:tHcy ratio: 1.6 [1.2-2.1] vs 1.8 [1.4-2.3] vs 2.0 [1.6-2.6]; *P* < .001), in those who currently smoke vs formerly or never smoked (tHCy: 1.677 [1.366-2.055] mg/dL vs 1.650 [1.379-2.042] mg/dL vs 1.758 [1.420-2.204] mg/dL; *P* = .003; Met:tHcy ratio: 1.9 [1.4-2.4] vs 1.9 [1.5-2.4] vs 1.8 [1.3-2.3]; *P* = .003), in those with inadequate levels of physical activity vs health-enhancing or fitness-enhancing levels of physical activity (tHCy: 1.839 [1.461-2.366] mg/dL vs 1.677 [1.379-2.069] mg/dL vs 1.582 [1.352-1.880] mg/dL; *P* < .001; Met:tHcy ratio: 1.7 [1.2-2.2] vs 1.8 [1.4-2.4] vs 2.0 [1.6-2.5]; *P* = .001), those with higher vs lower concentrations of CRP (tHCy: 1.650 [1.366-2.055] mg/dL vs 1.785 [1.461-2.177] mg/dL; *P* = .04; Met:tHcy ratio: 1.9 [1.5-2.4] vs 1.7 [1.3-2.2]; *P* = .02), those taking less than 4 drugs vs 4 or more drugs (tHCy: 1.690 [1.420-2.069] mg/dL vs 1.650 [1.312-2.096] mg/dL; *P* = .006; Met:tHcy ratio: 1.9 [1.5-2.4] vs 1.9 [1.4-2.5]; *P* = .004), and those not taking vs taking B vitamin supplements (tHCy: 1.542 [1.258-2.880] mg/dL vs 1.690 [1.393-2.109] mg/dL; *P* < .001; Met:tHcy ratio: 2.0 [1.5-2.6] vs 1.9 [1.54-2.4]; *P* < .001) (Table 1). We found a significantly higher concentration of tHcy and a lower Met:tHcy ratio among participants diagnosed with hypertension vs no hypertension (tHCy: 1.704 [1.406-2.137] mg/dL vs 1.623 [1.339-1.974] mg/dL; *P* = .008; Met:tHcy ratio: 1.8 [1.4-2.3] vs 2.0 [1.5-2.6]; *P* = .003), chronic kidney disease vs no chronic kidney disease (tHCy: 1.947 [1.596-2.515] mg/dL vs 1.596 [1.325-1.947] mg/dL; *P* < .001; Met:tHcy ratio: 1.6 [1.1-2.0] vs 2.0 [1.5-2.5]; *P* < .001), and those with a *MTHFR* 677 TT allele vs a *MTHFR *677 CT or CC allele (tHCy: 1.636 [1.352-2.015] mg/dL vs 1.690 [1.393-2.069] mg/dL vs 1.799 [1.488-2474] mg/dL; *P* < .001; Met:tHcy ratio: 1.9 [1.5-2.5] vs 1.8 [1.5-2.4] vs 1.8 [1.2-2.3]; *P* < .001). The median (IQR) concentration of Met was significantly lower among older participants vs younger participants (0.357 [0.305-0.417] mg/dL vs 0.329 [0.284-0.381] mg/dL; *P* < .001), women vs men (0.354 [0.306-0.414] mg/dL vs 0.314 [0.290-0.400] mg/dL; *P* < .001), and those with only elementary education vs those with high school or university educations (0.333 [0.288-0.387] mg/dL vs 0.339 [0.288-0.396] mg/dL vs 0.359 [0.312-0.421] mg/dL; *P* < .001).

**Table 1.** Serum Concentrations of tHcy and Met and Met:tHcy Ratio According to Baseline Sociodemographic, Lifestyle, Clinical, and Genetic Characteristics

Characteristic	tHcy, median (IQR), mg/dL	*P* value^a^	Met, median (IQR), mg/dL	*P* value^a^	Met:tHcy, median (IQR)	*P* value^a^
Age, y						
<78	1.596 (1.325-1.920)	<.001	0.357 (0.305-0.417)	<.001	2.0 (1.6-2.6)	<.001
≥78	1.893 (1.542-2.420)		0.329 (0.284-0.381)		1.6 (1.2-2.0)	
Sex						
Men	1.785 (1.515-2.164)	<.001	0.354 (0.306-0.414)	.02	1.8 (1.4-2.3)	<.001
Women	1.623 (1.312-2.042)		0.341 (0.290-0.400)		1.9 (1.5-2.5)	
Education						
Elementary	1.893 (1.555-2.393)	<.001	0.333 (0.288-0.387)	.01	1.6 (1.2-2.1)	<.001
High school	1.704 (1.393-2.137)		0.339 (0.288-0.396)		1.8 (1.4-2.3)	
University	1.596 (1.339-1.920)		0.359 (0.312-0.421)		2.0 (1.6-2.6)	
Smoking						
Never	1.677 (1.366-2.055)	.003	0.348 (0.293-0.406)	.15	1.9 (1.4-2.4)	.009
Former	1.650 (1.379-2.042)		0.350 (0.299-0.406)		1.9 (1.5-2.4)	
Current	1.758 (1.420-2.204)		0.341 (0.294-0.394)		1.8 (1.3-2.3)	
Alcohol consumption						
Never or occasional	1.785 (1.474-2.272)	.88	0.333 (0.288-0.391)	.55	1.7 (1.2-2.2)	.87
Light or moderate	1.650 (1.366-2.015)		0.354 (0.302-0.411)		1.9 (1.5-2.5)	
Heavy	1.623 (1.339-2.042)		0.348 (0.291-0.411)		1.9 (1.5-2.5)	
Physical activity						
Inadequate	1.839 (1.461-2.366)	<.001	0.338 (0.293-0.402)	.42	1.7 (1.2-2.2)	.001
Health-enhancing	1.677 (1.379-2.069)		0.348 (0.294-0.400)		1.8 (1.4-2.4)	
Fitness-enhancing	1.582 (1.352-1.880)		0.357 (0.302-0.411)		2.0 (1.6-2.5)	
BMI						
<18.5	1.609 (1.393-2.407)	.38	0.332 (0.280-0.424)	.77	1.8 (1.2-2.2)	.05
18.5-24.9	1.636 (1.339-2.028)		0.345 (0.294-0.405)		1.9 (1.5-2.5)	
25.0-29.9	1.690 (1.420-2.069)		0.351 (0.297-0.406)		1.9 (1.5-2.4)	
≥30	1.717 (1.447-2.137)		0.353 (0.299-0.405)		1.9 (1.4-2.3)	
CRP level, mg/L						
≤5	1.650 (1.366-2.055)	.04	0.350 (0.297-0.408)	.24	1.9 (1.5-2.4)	.02
>5	1.785 (1.461-2.177)		0.335 (0.291-0.394)		1.7 (1.3-2.2)	
Drugs, No.						
<4	1.690 (1.420-2.069)	.006	0.351 (0.299-0.408)	.76	1.9 (1.5-2.4)	.004
≥4	1.650 (1.312-2.096)		0.336 (0.290-0.397)		1.9 (1.4-2.5)	
Dyslipidemia						
Yes	1.677 (1.379-2.096)	.32	0.342 (0.296-0.402)	.32	1.9 (1.4-2.4)	.13
No	1.663 (1.366-2.069)		0.350 (0.296-0.406)		1.9 (1.4-2.5)	
Diabetes						
Yes	1.650 (1.393-2.123)	.68	0.353 (0.309-0.418)	.27	1.9 (1.5-2.5)	.02
No	1.677 (1.379-2.082)		0.347 (0.294-0.403)		1.9 (1.4-2.4)	
Hypertension						
Yes	1.704 (1.406-2.137)	.008	0.342 (0.294-0.399)	.08	1.8 (1.4-2.3)	.003
No	1.623 (1.339-1.974)		0.356 (0.299-0.418)		2.0 (1.5-2.6)	
Chronic kidney disease						
Yes	1.947 (1.596-2.515)	<.001	0.345 (0.291-0.393)	.27	1.6 (1.1-2.0)	<.001
No	1.596 (1.325-1.947)		0.348 (0.299-0.409)		2.0 (1.5-2.5)	
Use of antihypertensive drugs						
Yes	1.690 (1.352-2.096)	.45	0.344 (0.299-0.406)	.08	1.9 (1.4-2.4)	.11
No	1.677 (1.379-2.069)		0.348 (0.296-0.403)		1.9 (1.4-2.4)	
Use of B vitamin supplements						
Yes	1.542 (1.258-1.880)	<.001	0.333 (0.284-0.385)	.93	2.0 (1.5-2.6)	<.001
No	1.690 (1.393-2.109)		0.348 (0.296-0.406)		1.9 (1.4-2.4)	
*MTHFR* 677C>T polymorphism						
CC	1.636 (1.352-2.015)	<.001	0.348 (0.294-0.402)	.96	1.9 (1.5-2.5)	<.001
CT	1.690 (1.393-2.069)		0.345 (0.297-0.403)		1.8 (1.5-2.4)	
TT	1.799 (1.488-2.474)		0.353 (0.297-0.415)		1.8 (1.2-2.3)	

Abbreviations: BMI, body mass index (calculated as weight in kilograms divided by height in meters squared); CC, *MTHFR* 677CC polymorphism; CRP, C-reactive protein; CT, *MTHFR* 677CT polymorphism; IQR, interquartile range; Met, methionine; *MTHFR*, methylenetetrahydrofolate reductase; tHcy, homocysteine; TT, *MTHFR* 677TT polymorphism.

SI conversion factors: To convert Met to micromoles per liter, multiply by 67.02; tHcy to micromoles per liter, multiply by 7.397.

^a^ Linear regression with tHcy and Met as the dependent variable and with the rest of the variables in the left column of the table as independent variables.

**Figure 2.**
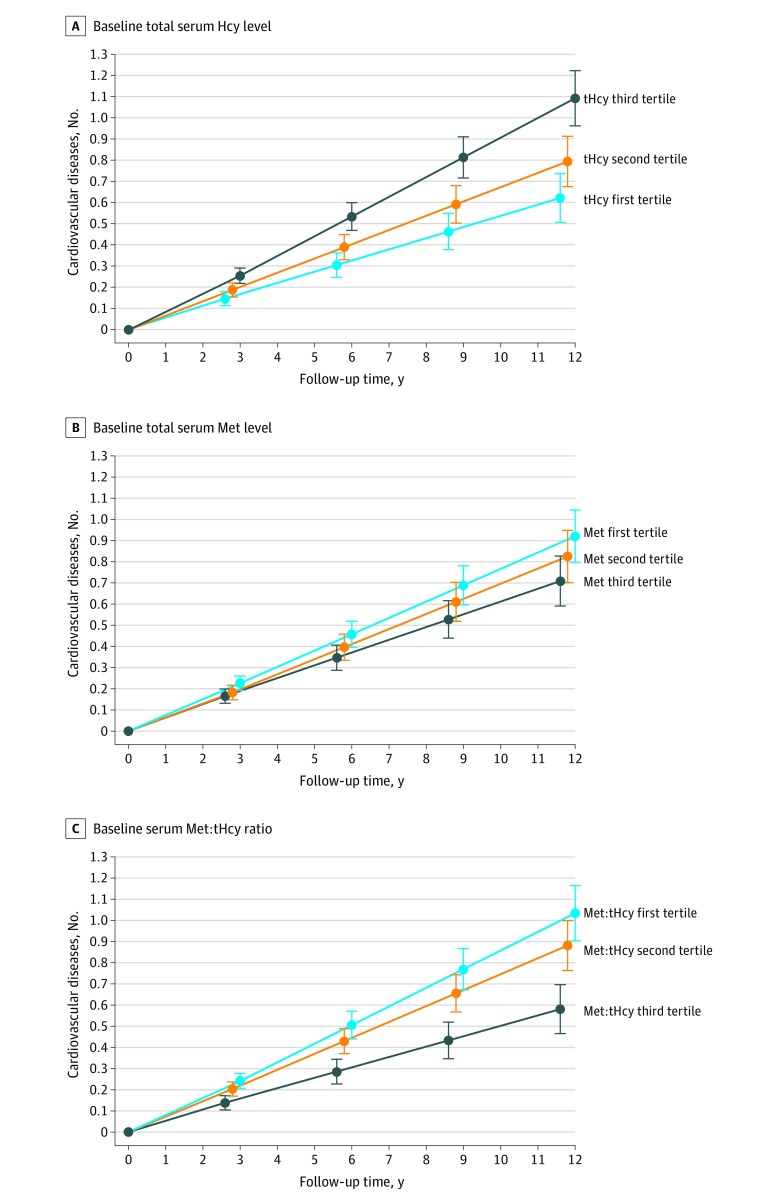
Estimated Rate of Cardiovascular Disease Accumulation During the 12-Year Follow-up by Baseline Concentrations of Homocysteine (tHcy), Methionine (Met), and the Met:tHcy Ratio Fully adjusted models were adjusted for age, sex, education, smoking habit, alcohol consumption, physical activity, body mass index, C-reactive protein level, number of drugs, dyslipidemia, diabetes, hypertension, chronic kidney disease, use of antihypertensive drugs, use of B vitamin supplements, and time of death or dropout. Tertiles for tHcy were defined as follows: first tertile, less than 1.49 mg/dL; second tertile, 1.50-1.91 mg/dL; third tertile, greater than 1.91 mg/dL. Tertiles for Met were defined as follows: first tertile, less than 0.31 mg/dL; second tertile, 0.31-0.38 mg/dL; third tertile, greater than 0.38 mg/dL. Tertiles for Met:tHcy ratio were defined as follows: first tertile, less than 1.6; second tertile, 1.6-2.2; third tertile, greater than 2.2. To convert Met to micromoles per liter, multiply by 67.02; tHcy to micromoles per liter, multiply by 7.397.

In the longitudinal analyses during the 12-year follow-up, tHcy showed a significant association with the rate of CV disease accumulation over time (β = 0.023 per year; 95% CI, 0.015 to 0.030; *P* < .001). In contrast, a significant inverse association was seen with Met (β = −0.007 per year; 95% CI, −0.013 to −0.001; *P* = .02) and with the Met:tHcy ratio (β = −0.017 per year; 95% CI, −0.023 to −0.011; *P* < .001) (Table 2). The analysis by tertiles showed a likely concentration-response association between the biomarkers and the rate of CV disease accumulation (highest vs lowest tertile of tHcy: β = 0.040 per year; 95% CI, 0.025 to 0.055; highest vs lowest tertile of Met: β = −0.017 per year; 95% CI, −0.032 to −0.002; highest vs lowest tertile of tHcy:Met ratio: β = −0.039 per year; 95% CI, −0.054 to −0.024) (Figure 2). *MTHFR* 677 C>T polymorphism was not significantly associated with the outcome in any of the models (eTable 3 in the Supplement). In the sensitivity analysis excluding 1 CV disease at a time from the main outcome variable, all models provided similar results (eg, ischemic heart disease, tHcy: β = 0.023 per year; 95% CI, 0.013 to 0.027; *P* < .001; Met: β = −0.006 per year; 95% CI, −0.011 to −0.0003; *P* = .04; Met:tHcy ratio: β = −0.015 per year; 95% CI, −0.020 to −0.009; *P* < .001) (eTable 4 in the Supplement).

**Table 2.** Association of Baseline Concentrations of tHcy, Met, and Met:tHcy Ratio With Annual Rate of Cardiovascular Disease Accumulation During the 12-Year Follow-up

Biomarker	Minimally adjusted model^a^		Fully adjusted model^b^	
	β coefficient (95% CI)^c^	*P* value	β coefficient (95% CI)^c^	*P* value
tHcy	0.017 (0.011 to 0.024)	<.001	0.023 (0.015 to 0.030)	<.001
Met	–0.006 (–0.012 to –0.0002)	.04	–0.007 (–0.013 to –0.001)	.02
Met:tHcy	–0.018 (–0.024 to –0.012)	<.001	–0.017 (–0.023 to –0.011)	<.001

Abbreviations: Met, methionine; tHcy, homocysteine.

^a^ Adjusted for age, sex, and education.

^b^ Adjusted by for age, sex, education, smoking habit, alcohol consumption, physical activity, body mass index, C-reactive protein level, number of drugs, dyslipidemia, diabetes, hypertension, chronic kidney disease, use of antihypertensive drugs, use of B vitamin supplements, and time of death or dropout.

^c^ β coefficient for 1 standard deviation change in each biomarker.

The associations between high concentrations of tHcy and the rate of CV disease accumulation were similar for both *MTHFR* genotypes (CC: β = 0.034 per year; 95% CI, 0.016-0.051; *P* < .001; CT/TT: β = 0.035 per year; 95% CI, 0.019-0.052; *P* < .001) (Table 3). For Met, a significant association was only seen in participants with a low concentration of Met and the CT/TT genotype (β = 0.023 per year; 95% CI, 0.006-0.041; *P* = .009) (Table 3; eFigure in the Supplement). These results were further corroborated by the positive association with the rate of CV multimorbidity development in participants with low Met:tHcy ratios and both the CC group (β = 0.039 per year; 95% CI, 0.021-0.057; *P* < .001) and the CT/TT group (β = 0.037 per year; 95% CI, 0.021-0.054; *P* < .001) (Table 3).

**Table 3.** Association of Baseline Concentrations of tHcy, Met, and Met:tHcy Ratio With the Rate of Cardiovascular Disease Accumulation During the 12-Year Follow-up Stratified by *MTHFR* 677C>T Polymorphism^a^

*MTHFR* polymorphism	tHcy				Met				Met:tHcy			
	Levels^b^	Participants, No.	β coefficient (95% CI)	*P* value	Levels^b^	Participants, No.	β coefficient (95% CI)	*P* value	Levels^b^	Participants, No.	β coefficient (95% CI)	*P* value
CC, wild type	Low	494	1 [Reference]	NA	High	443	1 [Reference]	NA	High	486	1 [Reference]	NA
	High	410	0.034 (0.016 to 0.051)	<.001	Low	461	0.012 (−0.010 to 0.024)	.20	Low	418	0.039 (0.021 to 0.057)	<.001
CT and TT	Low	432	0.012 (−0.005 to 0.029)	.17	High	449	0.007 (−0.008 to 0.025)	.43	High	428	0.014 (−0.003 to 0.030)	.11
	High	478	0.035 (0.019 to 0.052)	<.001	Low	461	0.023 (0.006 to 0.041)	.009	Low	482	0.037 (0.021 to 0.054)	<.001

Abbreviations: CC, *MTHFR* 677CC polymorphism; CT, *MTHFR* 677CT polymorphism; Met, methionine; *MTHFR*, methylenetetrahydrofolate reductase; tHcy, homocysteine; TT, *MTHFR* 677TT polymorphism.

^a^ Models adjusted for age, sex, education, smoking habit, alcohol consumption, physical activity, body mass index, C-reactive protein level, number of drugs, dyslipidemia, diabetes, hypertension, chronic kidney disease, use of antihypertensive drugs, use of B vitamin supplements, and time of death or dropout.

^b^ Levels for tHcy, Met, and Met:tHcy ratio established according to the median of the distribution (tHcy, 1.68 mg/dL [to convert to micromoles per liter, multiply by 7.397]; Met, 0.35 mg/dL [to convert to micromoles per liter, multiply by 67.02]; Met:tHcy ratio, 1.9).

## Discussion

In this longitudinal population-based study of older adults, a higher concentration of tHcy, a lower concentration of Met, and a lower Met:tHcy ratio (suggesting impaired methylation activity) were all associated with an increased rate of CV multimorbidity development during a 12-year period. Genetic predisposition (ie, the *MTHFR* 677T allele) further contributed to accelerated CV multimorbidity development in participants with low Met concentrations. Results were independent of sociodemographic, lifestyle, and clinical factors and did not seem to be driven by the incidental diagnosis of a specific CV disease but rather with the global burden of CV multimorbidity. Even if the effect sizes were relatively small, the biological coherence, the concentration-response associations, and the statistical robustness of the findings point to a potentially meaningful role of these biomarkers in the detection of CV multimorbidity and possibly also its etiology.

The prevalence of CV multimorbidity is increasing rapidly in the older population,^25^ and the combination of any of the conditions included in CV multimorbidity is associated with a multiplicative mortality risk.^26^ Moreover, CV multimorbidity is highly disabling in older adults and is associated with faster cognitive decline,^27,28^ thus affecting quality of life as well as the ability to live independently. Therefore, knowledge of the rate of CV multimorbidity development could help to target individuals who would benefit most from primary and secondary prevention of CV disease. In addition, if specific biological markers can be identified to capture CV aging, measuring these could track the efficacy of interventions aimed precisely at slowing CV dysfunction and decline. This will undoubtedly be the next phase of improvement in human longevity, for which CV diseases are still the leading threat.^29^

The pathophysiologic mechanisms underlying the increased risk of CV diseases in individuals with hyperhomocysteinemia have been comprehensively studied.^30,31^ Increased tHcy concentrations have consistently been shown to be associated with increased risk of new CV events,^32^ in line with our findings. Thus, in patients with CV diseases, tHcy concentrations could be used as a prognostic factor and for stricter surveillance of lifestyle and treatment.^9^ Other nonatherogenic mechanisms may explain the nonselective association between tHcy and CV disease accumulation described in our study. Indeed, hyperhomocysteinemia, which has been described to induce both ventricular systolic and diastolic dysfunction, has been associated with heart failure even in the absence of ischemic heart disease.^33,34^ The increased deposition of interstitial and perivascular collagen triggered by high tHcy concentrations may explain its effect on heart failure via nonatherosclerotic mechanisms. Moreover, telomere attrition in cardiomyocytes, potentially accelerated by higher tHcy blood concentrations, can promote faster aging of myocardium, inducing pump insufficiency and chambers dilation.^1,35,36^ Increased tHcy concentrations have been also associated with impaired autophagy of both cardiomyocytes and smooth vascular cells, leading to the accumulation of senescent cells and consequently organ dysfunction.^37,38^

Much less is known regarding the role and pathophysiology of Met. A possible pathogenic pathway may involve glycine N-methyltransferase (GNMT), the most abundant liver methyltransferase regulating the availability of the biologic methyl donor, S-adenosylmethionine. Met deficiency leads to reduced GNMT flux,^39^ which is linked to hepatic lipid accumulation, hyperlipidemia, and the deposition of oxidized LDL in vascular walls, all of which have been associated with increased risk of atherosclerosis.^11,12,40^ Another possible pathway is through the role of Met in methylation reactions. Met deficiency can induce site-specific hypomethylation,^41^ leading to dysfunctional epigenetic modifications described recently in the expression of proprotein convertase subtilisin/kexin type 9, a serine protease involved in the degradation of both hepatic and extrahepatic LDL receptors, thereby increasing circulating LDL cholesterol concentrations.^42^ Indeed, higher concentrations of Met and an improved methylation activity, as indicated by the Met:tHcy ratio, were both associated with a decreased rate of CV disease accumulation in our study, which is consistent with findings from animal experimental studies^11,12^ and a 2019 study examining the risk of dementia development and structural brain changes.^21^ Finally, Met deficiency may lead to deficiency of glutathione, a major intracellular antioxidant, which has been linked to metabolic and CV diseases.^43^

The *MTHFR* 677C>T polymorphism leads to a modest increase in tHcy. Individuals with the homozygous TT genotype have approximately 0.34 mg/dL higher tHcy levels than those with the wild-type CC variant.^44,45^ The difference depends mainly on folate status.^46^ Lack of power may therefore be the reason why most studies, including ours, have failed to show any difference in the risk of CV diseases in people with this polymorphism,^32^ but meta-analyses have revealed such an effect consistently, as first shown in 2002 by Klerk et al.^45^ It has recently been hypothesized that the *MTHFR* 677C>T polymorphism could influence CV pathology through mechanisms that are independent of tHcy, such as via blood pressure disorders.^47^ Interestingly, in our study, the association of low Met with the rate of CV multimorbidity development was restricted to the group with the CT/TT alleles of *MTHFR*, suggesting an interactive effect of both Met and genotype. Such a finding, together with the significant protective association of the Met:tHcy ratio in our models, suggests that we need to further examine methylation-derived modifications of gene expression in relation to CV multimorbidity.

### Strengths and Limitations

The main strength of our study is the use of a longitudinal population-based study in older adults with detailed clinical characterization and available data on potential confounders. The identification of CV diseases was done through a clinically driven algorithm, integrating different sources of data. Thus, the risk of misclassification and underdetection of the outcome was limited, as previously shown.^19^ Individuals with CV diseases at baseline were excluded from our analyses, which minimized the risk of reverse causality.

Several limitations should be considered. Time-varying measurements for the biomarkers (ie, the marker may not be constant over time) could lead to biased associations due to regression dilution. It has been shown that failure to correct for regression dilution may underestimate the relative risks of disease by 50% after 10 years.^48^ Moreover, blood measurements were performed under nonfasting conditions, which could have direct consequences on serum Met levels following a meal.^49^ However, in previous studies, adjustment for fasting status did not alter the association between plasma Met concentrations and acute myocardial infarction^14^ or other outcomes.^21^ The low number of participants with the TT genotype forced us to explore its association in combination with the CT genotype, even if the latter had tHcy concentrations that were closer to the CC rather than the TT genotype. Moreover, we could not control for confounding from ancestry or technical bias in genotyping. Even after adjustment for potential confounders, the possibility of residual confounding cannot be discarded. Furthermore, although this is a prospective study, reverse causality should not be ruled out given that elevated tHcy is associated with risk factors often targeted for treatment in relation to CV health.

## Conclusions

This study adds further epidemiologic evidence to the hypothesis that serum tHcy and Met concentrations and Met:tHcy ratio are important independent risk factors not only for the incidence of multiple CV diseases of diverse nature, but also for the rate at which these different CV diseases accumulate in older age. The interactive associations of Met concentrations and the *MTHFR* 677C>T polymorphism, together with the association found for the Met:tHcy ratio, point to the relevance of impaired methylation in the pathogenesis of CV aging but also call for further studies investigating alternative pathways.

## Notes

Supplement. **eTable 1. ***ICD-10* Codes and Additional Clinical and Drug-Related Parameters **eTable 2. **Descriptive Analysis of the Baseline SNAC-K Population and the Cardiovascular Disease-Free Study Sample, Stratified by Sex **eTable 3. **Association Between Baseline Concentrations of tHcy, Met, Met:tHcy Ratio (Continuous), *MTHFR* 677C>T Polymorphism and the Annual Rate of Cardiovascular Disease Accumulation During the 12-Year Follow-up **eTable 4. **Sensitivity Analysis: Association Between Baseline Concentrations of tHcy, Met and Met:tHcy Ratio (Continuous) and the Rate of Cardiovascular Disease Accumulation During the 12-Year Follow-up Removing 8 Cardiovascular Diseases Individually From Original Total **eFigure. **Estimated Rate of Cardiovascular Disease Accumulation During the 12-Year Follow-up by Baseline Concentrations of tHcy, Met, and Met:tHcy Ratio and *MTHFR* 677C>T Polymorphism
