# Supplementary material for: Association of Homocysteine, Methionine, and MTHFR 677C>T Polymorphism With Rate of Cardiovascular Multimorbidity Development in Older Adults in Sweden
Source: JAMA Netw Open. 2020 May 20;3(5):e205316. doi: 10.1001/jamanetworkopen.2020.5316 (PMC7240355; doi:10.1001/jamanetworkopen.2020.5316)
Supplement: Supplement. — eTable 1. ICD-10 Codes and Additional Clinical and Drug-Related Parameters eTable 2. Descriptive Analysis of the Baseline SNAC-K Population and the Cardiovascular Disease-Free Study Sample, Stratified by Sex eTable 3. Association Between Baseline Concentrations of tHcy, Met, Met:tHcy Ratio (Continuous), MTHFR 677C>T Polymorphism and the Annual Rate of Cardiovascular Disease Accumulation During the 12-Year Follow-up eTable 4. Sensitivity Analysis: Association Between Baseline Concentrations of tHcy, Met and Met:tHcy Ratio (Continuous) and the Rate of Cardiovascular Disease Accumulation During the 12-Year Follow-up Removing 8 Cardiovascular Diseases Individually From Original Total eFigure. Estimated Rate of Cardiovascular Disease Accumulation During the 12-Year Follow-up by Baseline Concentrations of tHcy, Met, and Met:tHcy Ratio and MTHFR 677C>T Polymorphism [file jamanetwopen-3-e205316-s001.pdf]

Supplementary Online Content

Calderón-Larrañaga A, Saadeh M, Hooshmand B, et al. Association of homocysteine, methionine, and *MTHFR* 677C>T polymorphism with rate of cardiovascular multimorbidity development in older adults in Sweden. *JAMA Netw Open*. 2020;3(5):e205316. doi:10.1001/jamanetworkopen.2020.5316

eTable 1. *ICD-10* Codes and Additional Clinical and Drug-Related Parameters
eTable 2. Descriptive Analysis of the Baseline SNAC-K Population and the Cardiovascular Disease-Free Study Sample, Stratified by Sex
eTable 3. Association Between Baseline Concentrations of tHcy, Met, Met:tHcy Ratio (Continuous), *MTHFR* 677C>T Polymorphism and the Annual Rate of Cardiovascular Disease Accumulation During the 12-Year Follow-up
eTable 4. Sensitivity Analysis: Association Between Baseline Concentrations of tHcy, Met and Met:tHcy Ratio (Continuous) and the Rate of Cardiovascular Disease Accumulation During the 12-Year Follow-up Removing 8 Cardiovascular Diseases Individually From Original Total
eFigure. Estimated Rate of Cardiovascular Disease Accumulation During the 12-Year Follow-up by Baseline Concentrations of tHcy, Met, and Met:tHcy Ratio and *MTHFR* 677C>T Polymorphism

This supplementary material has been provided by the authors to give readers additional information about their work.

© 2020 Calderón-Larrañaga A et al. *JAMA Network Open.*

eTable 1. *ICD-10* Codes and Additional Clinical and Drug-Related Parameters

eTable 1A. Descriptors of ICD-10 codes included and excluded in each cardiovascular disease category

ATRIAL FIBRILLATION	
Included ICD-10 codes and labels	
I48	Atrial fibrillation and flutter
BRADYCARDIAS AND CONDUCTION DISEASES	
Included ICD-10 codes and labels	
I441	Atrioventricular block, second degree
I442	Atrioventricular block, complete
I443	Other and unspecified atrioventricular block
I453	Trifascicular block
I455	Other specified heart block
Z950	Presence of cardiac pacemaker
CARDIAC VALVE DISEASES	
Included ICD-10 codes and labels	
I05	Rheumatic mitral valve diseases
I06	Rheumatic aortic valve diseases
I07	Rheumatic tricuspid valve diseases
I08	Multiple valve diseases
I091	Rheumatic diseases of endocardium, valve unspecified
I098	Other specified rheumatic heart diseases
I34	Nonrheumatic mitral valve disorders
I35	Nonrheumatic aortic valve disorders
I36	Nonrheumatic tricuspid valve disorders
I37	Pulmonary valve disorders
I38	Endocarditis, valve unspecified
I390	Mitral valve disorders in diseases classified elsewhere
I391	Aortic valve disorders in diseases classified elsewhere
I392	Tricuspid valve disorders in diseases classified elsewhere
I393	Pulmonary valve disorders in diseases classified elsewhere
I394	Multiple valve disorders in diseases classified elsewhere
Q22	Congenital malformations of pulmonary and tricuspid valves
Q23	Congenital malformations of aortic and mitral valves
Z952	Presence of prosthetic heart valve
Z953	Presence of xenogenic heart valve
Z954	Presence of other heart-valve replacement
CEREBROVASCULAR DISEASE	
Included ICD-10 codes and labels	
G45	Transient cerebral ischaemic attacks and related syndromes
G46	Vascular syndromes of brain in cerebrovascular diseases
I60	Subarachnoid haemorrhage
I61	Intracerebral haemorrhage
I62	Other nontraumatic intracranial haemorrhage
I63	Cerebral infarction
I64	Stroke, not specified as haemorrhage or infarction
I67	Other cerebrovascular diseases
I69	Sequelae of cerebrovascular disease
HEART FAILURE	
Included ICD-10 codes and labels	
I110	Hypertensive heart disease with (congestive) heart failure
I130	Hypertensive heart and renal disease with (congestive) heart failure
I132	Hypertensive heart and renal disease with both (congestive) heart failure and renal failure
I27	Other pulmonary heart diseases

© 2020 Calderón-Larrañaga A et al. *JAMA Network Open.*

I280	Arteriovenous fistula of pulmonary vessels
I42	Cardiomyopathy
I43	Cardiomyopathy in diseases classified elsewhere
I50	Heart failure
I515	Myocardial degeneration
I517	Cardiomegaly
I528	Other heart disorders in other diseases classified elsewhere
Z941	Heart transplant status
Z943	Heart and lungs transplant status
ISCHEMIC HEART DISEASE	
Included ICD-10 codes and labels	
I20	Angina pectoris
I21	Acute myocardial infarction
I22	Subsequent myocardial infarction
I24	Other acute ischaemic heart diseases
I25	Chronic ischaemic heart disease
Z951	Presence of aortocoronary bypass graft
Z955	Presence of coronary angioplasty implant and graft
OTHER CARDIOVASCULAR DISEASES	
Included ICD-10 codes and labels	
I09	Other rheumatic heart diseases
I281	Aneurysm of pulmonary artery
I310	Chronic adhesive pericarditis
I311	Chronic constrictive pericarditis
I456	Pre-excitation syndrome
I495	Sick sinus syndrome
I498	Other specified cardiac arrhythmias
I70	Atherosclerosis
I71	Aortic aneurysm and dissection
I72	Other aneurysm and dissection
I790	Aneurysm of aorta in diseases classified elsewhere
I791	Aortitis in diseases classified elsewhere
I950	Idiopathic hypotension
I951	Orthostatic hypotension
I958	Other hypotension
Q20	Congenital malformations of cardiac chambers and connections
Q21	Congenital malformations of cardiac septa
Q24	Other congenital malformations of heart
Q25	Congenital malformations of great arteries
Q26	Congenital malformations of great veins
Q27	Other congenital malformations of peripheral vascular system
Q28	Other congenital malformations of circulatory system
Z958	Presence of other cardiac and vascular implants and grafts
Z959	Presence of cardiac and vascular implant and graft, unspecified
Excluded ICD-10 codes and labels	
I091	Rheumatic diseases of endocardium, valve unspecified
I098	Other specified rheumatic heart diseases
I702	Atherosclerosis of arteries of extremities
PERIPHERAL VASCULAR DISEASE	
Included ICD-10 codes and labels	
I702	Atherosclerosis of arteries of extremities
I73	Other peripheral vascular diseases
I792	Peripheral angiopathy in diseases classified elsewhere

© 2020 Calderón-Larrañaga A et al. *JAMA Network Open.*

I798	Other disorders of arteries, arterioles and capillaries in diseases classified elsewhere
Excluded ICD-10 codes and labels	
I731	Thromboangiitis obliterans [Buerger]
I738	Other specified peripheral vascular diseases

eTable 1B. Additional clinical and drug-related parameters used for specific cardiovascular conditions

**Condition**	Clinical and drug-related parameters
**Atrial fibrillation**	Discrete P wave undetectable and irregular ventricular rate (12-lead electrocardiogram)
**Bradycardias and conduction diseases**	Presence of a cardiac pacemaker (12-lead electrocardiogram)
**Ischemic heart disease**	Use of organic nitrates (C01DA) or ranolazine (C01EB18)
**Peripheral vascular disease**	Use of cilostazol (B01AC23)

NOTE: The ATC codes corresponding to each drug are shown in brackets. The criteria presented in this table were used in addition to the cardiovascular diagnoses assigned in SNAC-K. For example, use of nitrates was considered to indicate presence of ischemic heart disease, even in the absence of other diagnostic information.

© 2020 Calderón-Larrañaga A et al. *JAMA Network Open.*

eTable 2. Descriptive Analysis of the Baseline SNAC-K Population and the Cardiovascular Disease-Free Study Sample, Stratified by Sex

	SNAC-K population*			Study sample§		
	**Total (N=3363)**	**Males (n=1181)**	**Females (n=2182)**	**Total (N=1969)**	**Males (n=708)**	**Females (n=1261)**
**Age, mean (SD)**	74.7 (11.2)	71.8 (10.1)	76.3 (11.5)	70.9 (9.8)	69.0 (8.9)	72.0 (10.0)
**Age groups n (%)**						
<78	1782 (53.0)	758 (64.2)	1024 (46.9)	1331 (67.6)	538 (76.0)	793 (62.9)
≥78	1581 (47.0)	423 (35.8)	1158 (53.1)	638 (32.4)	170 (24.0)	468 (37.1)
**Education n (%)**						
Elementary	590 (17.7)	168 (14.3)	422 (19.6)	265 (13.5)	84 (11.9)	181 (14.4)
High school	1651 (49.6)	495 (42.0)	1156 (53.7)	944 (48.0)	287 (40.6)	657 (52.1)
University	1090 (32.7)	516 (43.8)	574 (26.7)	759 (38.6)	336 (47.5)	423 (33.5)
**Smoking habit n (%)**						
Never	1553 (47.6)	408 (34.9)	1145 (54.7)	891 (45.5)	245 (34.8)	646 (51.6)
Former	1243 (38.1)	583 (49.9)	660 (31.2)	756 (38.6)	336 (47.7)	420 (33.5)
Current	465 (14.3)	178 (15.2)	287 (13.7)	311 (15.9)	124 (17.6)	187 (14.9)
**Alcohol consumption n (%)**						
Never/occasional	1229 (37.6)	284 (24.3)	945 (45.0)	568 (28.9)	143 (20.3)	425 (33.4)
Light/moderate	1527 (46.8)	759 (65.0)	768 (36.6)	1039 (52.9)	486 (68.8)	553 (44.0)
Heavy	510 (15.6)	124 (10.6)	386 (18.4)	357 (18.2)	77 (10.9)	280 (22.3)
**Physical activity n (%)**						
Inadequate	1163 (34.6)	347 (29.4)	816 (37.4)	467 (23.7)	171 (24.2)	296 (23.5)
Health-enhancing	1537 (45.7)	539 (45.6)	998 (45.7)	994 (50.5)	333 (47.0)	661 (52.4)
Fitness-enhancing	663 (19.7)	295 (25.0)	368 (16.9)	508 (25.8)	204 (28.8)	304 (24.1)
**BMI n (%)**						
Underweight	89 (2.9)	20 (1.8)	69 (3.6)	37 (1.9)	8 (1.2)	29 (2.4)
Normal weight	1364 (44.8)	433 (37.8)	931 (49.1)	851 (44.5)	264 (37.9)	587 (48.3)
Overweight	1200 (39.3)	537 (46.9)	663 (34.9)	779 (40.7)	340 (48.9)	439 (36.1)
Obese	390 (12.8)	155 (13.5)	235 (12.4)	245 (12.8)	84 (12.1)	161 (13.2)
**CRP n (%)**						
≤5 mg/L	2422 (79.5)	889 (79.9)	1533 (79.2)	1592 (82.4)	583 (83.3)	1009 (81.9)
>5 mg/L	626 (20.5)	224 (20.1)	402 (20.8)	340 (17.6)	117 (16.7)	223 (18.1)
**Number of drugs, mean (SD)**	4.0 (3.4)	3.2 (3.2)	4.5 (3.4)	2.9 (2.8)	2.0 (2.4)	3.4 (2.9)
**Dyslipidemia n (%)**	1558 (46.3)	505 (42.8)	1053 (48.3)	994 (50.5)	319 (45.1)	675 (53.5)
**Diabetes n (%)**	296 (8.8)	149 (12.6)	147 (6.7)	123 (6.25)	66 (9.3)	57 (4.5)
**Hypertension n (%)**	2277 (67.7)	797 (67.5)	1480 (67.8)	1342 (68.2)	481 (67.9)	861 (68.3)
**Chronic kidney disease n (%)**	1117 (33.2)	265 (22.4)	852 (39.1)	529 (26.9)	101 (14.3)	428 (33.9)
**Use of antihypertensive drugs n (%)**	943 (28.2)	342 (29.0)	601 (27.7)	367 (18.7)	119 (16.8)	248 (19.7)
**Use of B vitamin supplements n (%)**	512 (15.2)	125 (10.6)	387 (17.7)	202 (10.3)	49 (6.9)	153 (12.2)
**tHcy, mean (SD), µmol/L**	14.6 (7.1)	15.3 (7.2)	14.1 (7.0)	24.0 (6.1)	24.6 (6.1)	23.8 (6.1)
**Met, mean (SD), µmol/L**	23.8 (6.1)	24.1 (6.1)	23.5 (6.2)	13.6 (6.0)	14.6 (6.5)	13.1 (5.7)
**Met:tHcy, mean (SD)**	1.9 (0.8)	1.8 (0.7)	1.9 (0.8)	2.0 (0.8)	1.9 (0.7)	2.0 (0.8)
***MTHFR* n (% with)**						
CC	1387 (50.2)	512 (49.9)	875 (50.4)	904 (49.8)	324 (49.5)	580 (50.0)
CT	1110 (40.2)	397 (38.9)	713 (41.0)	734 (40.5)	255 (38.9)	479 (41.3)
TT	267 (9.7)	117 (11.4)	150 (8.6)	176 (9.7)	76 (11.6)	100 (8.6)

*Missing information: education 32; smoking habit 102; alcohol consumption 97; BMI 320; CRP 315; number of drugs 15; use of antihypertensive drugs 15; MTHFR 599.

§Missing information: education 1; smoking habit 11; alcohol consumption 5; BMI 57; CRP 37; number of drugs 5; use of antihypertensive drugs 5; MTHFR 155.

© 2020 Calderón-Larrañaga A et al. *JAMA Network Open.*

Abbreviations. CV: cardiovascular; SD: standard deviation; BMI: body mass index; CRP: C-reactive protein; MTHFR: Methylene tetrahydrofolate reductase; CC: *MTHFR*-677CC polymorphism; CT: *MTHFR*-677CT polymorphism; TT: *MTHFR*-677TT polymorphism.

© 2020 Calderón-Larrañaga A et al. *JAMA Network Open*.

eTable 3. Association Between Baseline Concentrations of tHcy, Met, Met:tHcy Ratio (Continuous), *MTHFR* 677C>T Polymorphism and the Annual Rate of Cardiovascular Disease Accumulation During the 12-Year Follow-up

	tHcy				Met				Met:tHcy			
	**Minimally adjusted**		**Fully adjusted**		**Minimally adjusted**		**Fully adjusted**		**Minimally adjusted**		**Fully adjusted**	
	**β-coef**	**95% CI**	**β-coef**	**95% CI**	**β-coef**	**95% CI**	**β-coef**	**95% CI**	**β-coef**	**95% CI**	**β-coef**	**95% CI**
**Without including *MTHFR* polymorphism in the models**												
**Biomarker**	0.017	0.011; 0.024	0.023	0.015; 0.031	-0.006	-0.012; -0.0002	-0.007	-0.013; -0.001	-0.018	-0.024; -0.012	-0.017	-0.023; -0.011
**Including *MTHFR* polymorphism in the models**												
**Biomarker**	0.017	0.010; 0.024	0.022	0.014; 0.031	-0.006	-0.012; -0.00007	-0.007	-0.013; -0.0007	-0.018	-0.025; -0.012	-0.017	-0.024; -0.011
**Polymorphism**												
**CT vs. CC**	-0.004	-0.033; 0.025	-0.004	-0.034; 0.026	-0.003	-0.032; 0.026	-0.003	-0.033; 0.027	-0.004	-0.033; 0.025	-0.004	-0.034; 0.026
**TT vs. CC**	-0.012	-0.061; 0.037	-0.006	-0.057; 0.045	-0.005	-0.053; 0.044	-0.0009	-0.051; 0.050	-0.007	-0.055; 0.041	-0.002	-0.053; 0.048

β coefficient for 1-standard deviation change in each biomarker.

Minimally adjusted models: adjusted by age, sex, education.

Fully adjusted models: additionally adjusted by smoking habit, alcohol consumption, physical activity, BMI, CRP, number of drugs, dyslipidemia, diabetes, hypertension, chronic kidney disease, use of antihypertensive drugs, use of B vitamin supplements, and time of death/drop-out.

Abbreviations. CV: cardiovascular; tHcy: homocysteine; Met: methionine; BMI: body mass index; CRP: C-reactive protein; MTHFR: Methylene tetrahydrofolate reductase; CC: *MTHFR*-677CC polymorphism; CT: *MTHFR*-677CT polymorphism; TT: *MTHFR*-677TT polymorphism.

© 2020 Calderón-Larrañaga A et al. *JAMA Network Open.*

eTable 4. Sensitivity Analysis: Association Between Baseline Concentrations of tHcy, Met and Met:tHcy Ratio (Continuous) and the Rate of Cardiovascular Disease Accumulation During the 12-Year Follow-up Removing 8 Cardiovascular Diseases Individually From Original Total

	tHcy			Met			Met:tHcy		
	β coefficient	95% CI	p-value	β coefficient	95% CI	p-value	β coefficient	95% CI	p-value
**Atrial fibrillation**	0.017	0.010; 0.023	<0.001	-0.005	-0.010; 0.0001	0.05	-0.013	-0.018; -0.008	<0.001
**Bradycardia conduction diseases**	0.023	0.014; 0.030	<0.001	-0.007	-0.013; -0.001	0.02	-0.017	-0.023; -0.011	<0.001
**Cardiac valve disease**	0.021	0.014; 0.028	<0.001	-0.007	-0.012; -0.002	0.01	-0.017	-0.022; -0.011	<0.001
**Cerebrovascular diseases**	0.019	0.012; 0.026	<0.001	-0.006	-0.012; -0.001	0.02	-0.015	-0.020; -0.009	<0.001
**Heart failure**	0.015	0.008; 0.021	<0.001	-0.005	-0.009; -0.0004	0.07	-0.012	-0.017; -0.007	<0.001
**Ischemic heart disease**	0.023	0.013; 0.027	<0.001	-0.006	-0.011; -0.0003	0.04	-0.015	-0.020; -0.009	<0.001
**Other CV diseases**	0.022	0.015; 0.029	<0.001	-0.006	-0.011; -0.001	0.03	-0.016	-0.022; -0.011	<0.001
**Peripheral vascular disease**	0.022	0.014; 0.029	<0.001	-0.006	-0.012; -0.0005	0.03	-0.017	-0.022; -0.011	<0.001

β coefficient for 1-standard deviation change in each biomarker.

Fully adjusted models: adjusted by age, sex, education, smoking habit, alcohol consumption, physical activity, BMI, CRP, number of drugs, dyslipidemia, diabetes, hypertension, chronic kidney disease, use of antihypertensive drugs, use of B vitamin supplements, and time of death/drop-out.

Abbreviations. CV: cardiovascular; tHcy: homocysteine; Met: methionine; BMI: body mass index; CRP: C-reactive protein.

© 2020 Calderón-Larrañaga A et al. *JAMA Network Open.*

eFigure. Estimated Rate of Cardiovascular Disease Accumulation During the 12-Year Follow-up by Baseline Concentrations of tHcy, Met, and Met:tHcy Ratio and *MTHFR* 677C>T Polymorphism

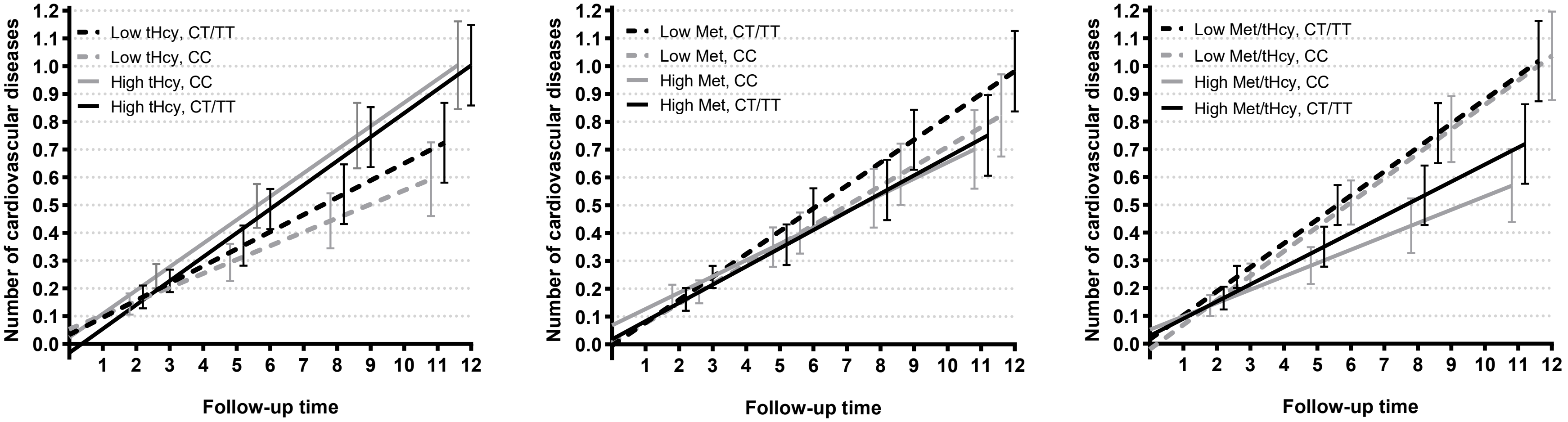

Fully adjusted models: adjusted by age, sex, education, smoking habit, alcohol consumption, physical activity, BMI, CRP, number of drugs, dyslipidemia, diabetes, hypertension, chronic kidney disease, use of antihypertensive drugs, use of B vitamin supplements, and time of death/drop-out.

Concentrations (high/low) of tHcy, Met and Met:tHcy ratio established according to the median of the distribution.

Median value for tHcy=12.4 µmol/L

Median value for Met=23.3 µmol/L

Median value for Met:tHcy=1.9

Abbreviations. CV: cardiovascular; tHcy: homocysteine; Met: methionine; BMI: body mass index; CRP: C-reactive protein; MTHFR: Methylene tetrahydrofolate reductase; CC: *MTHFR*-677CC polymorphism; CT: *MTHFR*-677CT polymorphism; TT: *MTHFR*-677TT polymorphism.

© 2020 Calderón-Larrañaga A et al. *JAMA Network Open.*
